# Alpha-Glucosidase Enzyme Biosensor for the Electrochemical Measurement of Antidiabetic Potential of Medicinal Plants

**DOI:** 10.1186/s11671-016-1292-1

**Published:** 2016-02-18

**Authors:** M. Mohiuddin, D. Arbain, A. K. M. Shafiqul Islam, M. S. Ahmad, M. N. Ahmad

**Affiliations:** Production Department, Palash Urea Fertilizer Factory Ltd., Bangladesh Chemical Industries Corporation, Dhaka, Bangladesh; School of Bioprocess Engineering, Universiti Malaysia Perlis, 01000 Kangar, Perlis Malaysia; Centre of Excellence for Advanced Sensor Technology, Universiti Malaysia Perlis, 01000 Kangar, Perlis Malaysia

**Keywords:** Electrochemical measurement, α-Glucosidase, Medicinal plants, Inhibition activity, Antidiabetic potential, Multi-walled carbon nanotubes (MWCNTs)

## Abstract

A biosensor for measuring the antidiabetic potential of medicinal plants was developed by covalent immobilization of α-glucosidase (AG) enzyme onto amine-functionalized multi-walled carbon nanotubes (MWCNTs-NH_2_). The immobilized enzyme was entrapped in freeze-thawed polyvinyl alcohol (PVA) together with *p*-nitrophenyl-α-d-glucopyranoside (PNPG) on the screen-printed carbon electrode at low pH to prevent the premature reaction between PNPG and AG enzyme. The enzymatic reaction within the biosensor is inhibited by bioactive compounds in the medicinal plant extracts. The capability of medicinal plants to inhibit the AG enzyme on the electrode correlates to the potential of the medicinal plants to inhibit the production of glucose from the carbohydrate in the human body. Thus, the inhibition indicates the antidiabetic potential of the medicinal plants. The performance of the biosensor was evaluated to measure the antidiabetic potential of three medicinal plants such as Tebengau (*Ehretis laevis*), Cemumar (*Micromelum pubescens*), and Kedondong (*Spondias dulcis*) and acarbose (commercial antidiabetic drug) via cyclic voltammetry, amperometry, and spectrophotometry. The cyclic voltammetry (CV) response for the inhibition of the AG enzyme activity by Tebengau plant extracts showed a linear relation in the range from 0.423–8.29 μA, and the inhibition detection limit was 0.253 μA. The biosensor exhibited good sensitivity (0.422 μA/mg Tebengau plant extracts) and rapid response (22 s). The biosensor retains approximately 82.16 % of its initial activity even after 30 days of storage at 4 °C.

## Background

Diabetes mellitus (DM) has become a worldwide epidemic. The International Diabetes Federation (IDF) reported that the number of adult diabetic patients worldwide is 366 million in November 2011, and it is predicted to increase to 552 million by 2030 [1]. DM is broadly classified into two categories, namely, type 1 and type 2 [2] in which about 90–95 % of diabetic patients suffer from type 2 DM [3]. Type 1 DM is due to inability of pancreas to produce insulin; therefore, patients with type 1 DM need regular insulin injection. Type 2 DM is caused by degradation of secreted insulin. This type of DM can be managed by diet control and consumption of various synthetic antidiabetic drugs [4]. Despite their efficacy, commercial antidiabetic drugs are often associated with some undesirable adverse side effects such as flatulence, diarrhea, abdominal pain, dropsy, drug-resistance, weight gain, and heart failure [5–8]. Besides, the synthetic drugs are expensive and have poor availability to many rural populations, particularly in developing countries [9]. As a result, the scientific investigation is increasing for the antidiabetic drugs from natural resources with minimal side effects and cheap.

The common method to determine the antidiabetic potential of medicinal plants is by measuring their ability to slow down the production of glucose from carbohydrate in the human body. In the laboratory, this is normally measured by their potency to inhibit the α-glucosidase (AG) enzyme or α-amylase enzyme reaction. This measurement is based on the rationale that inhibition of any of these enzymes would lower the glucose production. The measurement is usually performed through several conventional methods such as colorimetric [10], titration [11], high-performance liquid chromatography (HPLC) [12], and gas chromatography mass spectroscopy (GC-MS) methods [13]. However, these conventional methods have prominent drawbacks associated with extraction, sample preparation, intensive solvent usage, intensive labor, expensive devices, and time consumption. Moreover, they need a well-trained operator [14]. Hence, there is a demand to find an alternative for a practical and fast-response method suitable for fast screening the antidiabetic potential of medicinal plants.

Electrochemical techniques are widely used for analysis because of their fast response, low cost, good sensitivity, simplicity, and reproducibility as compared to conventional techniques [15]. Moreover, electrochemical instrumentation is amenable for mass production of sensors.

Suna Timur and Ulku Anik in 2007 [16] developed a biosensor using AG enzyme based on bismuth film electrode for inhibition detection of commercial antidiabetic drugs. The authors have not shown any extension of their sensor for measurement of antidiabetic potential of medicinal plants. Moreover, *p*-nitrophenyl-α-d-glucopyranoside (PNPG) was used as substrate in the solution but did not immobilize with AG enzyme for biosensor. El-Ries and his co-researcher in 2008 [17] determined the antidiabetic drugs repaglinide using carbon paste and glassy carbon electrodes by cyclic voltammetry (CV) and DPV method. The measurement relies on the electroactive properties of repaglinide and thus is not necessarily suitable for measurement of the antidiabetic properties of other medicinal plants.

In combination with electrochemical technique, multi-walled carbon nanotubes (MWCNTs) are used as carbon nanostructure materials for modification of biosensor due to their fascinating electrical, chemical, and mechanical properties, such as electrocatalytic outcome, rapid electron transfer rate, and broad working surface area [18]. Besides, chemical functionalization makes them particularly fascinating for electrochemical sensing [19]. As a result, MWCNTs contribute to enhance the electron transfer reaction for a wide range of molecules and biological species in electrochemical investigations [20].

In the present study, AG enzyme was covalently immobilized on MWCNTs-NH_2_ using 1-ethyl-3-(3-dimethylaminopropyl) carbodiimide (EDC) as coupling reagent associated to *N*-hydroxysuccinimide (NHS) in order to improve the immobilization efficiency for the electrochemical measurements of the antidiabetic potential of medicinal plants. The immobilized AG enzyme together with PNPG as substrate was subsequently entrapped within polyvinyl alcohol (PVA) and drop-coated on the screen-printed carbon electrode (SPCE) to develop the biosensor. PVA is a non-toxic, biologically compatible, and readily available low-cost synthetic polymer. It also has impact strength and good forming properties. So, PVA has extensively been used as a matrix for immobilization of enzymes [21]. The enzyme biosensor effectively measured the antidiabetic potential of medicinal plants with a great speed and sensitivity competitive with many of the others methods, suggesting its application in the rapid screening of medicinal plants for natural antidiabetic drugs.

### Detection Principle

**Fig. 1:**
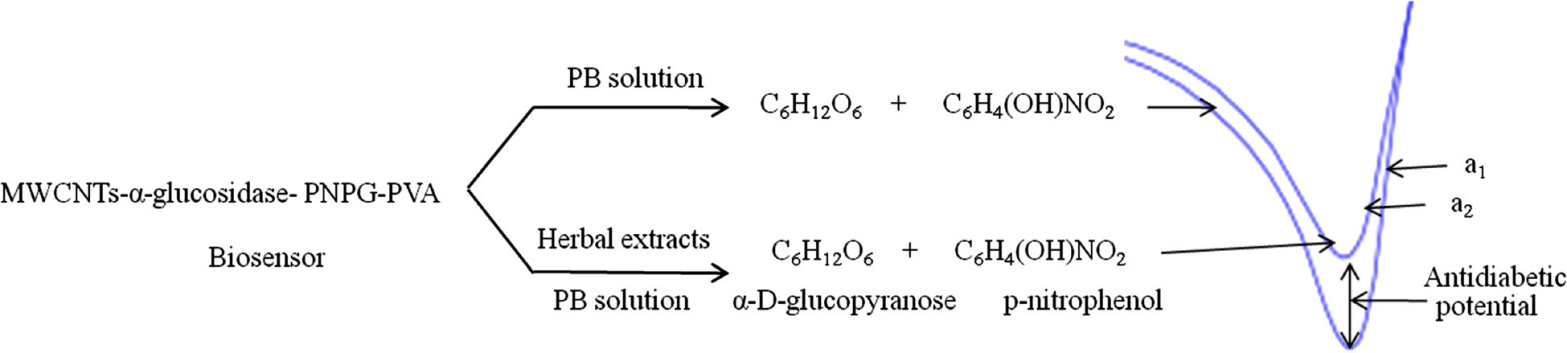
Reaction principle for the measurement of antidiabetic potential of medicinal plant extracts using biosensor via CV method

The antidiabetic potential of medicinal plant extracts and commercial drugs were measured by their ability to inhibit the AG enzymes and PNPG hydrolysis reaction which is shown in Fig. 1.

PNPG is hydrolyzed by the AG enzyme in the presence of 0.1 M phosphate buffer (PB) in the biosensor to release *p*-nitrophenol (p-NP) (Fig. 1). The liberated p-NP is electrochemically measured by CV method. A medicinal plant extract or synthetic antidiabetic drug inhibits this hydrolysis reaction and thus reduces the amount of liberated p-NP. The difference of p-NP liberated from the uninhibited (a_1_) and inhibited (a_2_) reactions reflects the antidiabetic potential of the medicinal plant extracts or the drug. The reduction peak of p-NP is used to measure the antidiabetic potential in this reaction principle.

## Methods

### Apparatus and Electrodes

Electrochemical measurements were conducted by cyclic voltammetric and amperometric method using a portable μStat 200 potentiostat controlled by Drop View 1.3 software equipped with three electrode configuration which was purchased from DropSens, Spain. A specific DropSens connector (ref. DRP-DSC) allowed the connection of the SPCEs (biosensor) to the potentiostat. These SPCEs consist of a conventional three-electrode configuration, printed on ceramic substrates. Both working and counter electrodes are made of carbon inks, whereas pseudo-reference electrode and electric contacts are made of silver. A working area constitutes the reservoir of the electrochemical cell with an actual volume of 50 μL.

A Shimadzu FT-IR spectrophotometer was used for characterization of developed biosensor. The spectra were recorded in the wave number range of 500–4000 cm^−1^ using 1:20 ratios of sample and potassium bromide (KBr) in the preparation of pellets. Scanning electron microscope (SEM) was used for characterization of immobilized AG enzyme on MWCNTs [22]. The colorimetric measurements were performed using a Lambda 25 UV–VIS spectrometer (PerkinElmer, USA).

### Chemicals and Reagents

α-Glucosidase (AG) enzyme, *para*-nitrophenol (granular), anhydrous sodium carbonate (Na_2_CO_3_), *p*-nitrophenyl-α-d-glucopyranoside (PNPG), 1-ethyl-3-(3-dimethylaminopropyl) carbodiimide (EDC) and *N*-hydroxy succinimide (NHS), poly-vinyl alcohol (PAV), ethylene diamine (EDA), *N*,*N*-dimethylformamide (DMF), sodium nitrite (NaNO_2_), and sulfuric acid (H_2_SO_4_) were obtained from Sigma-Aldrich. Multi-walled carbon nanotubes (MWCNTs, purity by weight 93 %) with average diameter 10–40 nm and length 1–25 μm were purchased from Fibermax Composites, Greece. Potassium chloride (KCl) was used as a supporting electrolyte. Di-potassium hydrogen phosphate (K_2_HPO_4_) and potassium dihydrogen phosphate (KH_2_PO_4_) were used for preparation of 0.1 M phosphate buffer (PB) solution. All other chemicals were used according to their analytical grade. Acarbose tablets (50 mg, Bayer, Germany) were obtained from a local pharmacy.

### Preparation of Herbal Plant Extracts

Tebengau (*Ehretis laevis*), Cemumar (*Micromelum pubescens*), and Kedondong (*Spondias dulcis*) plant leaves were collected from Agrotechnology Research Station in Sungai Chuchuh, Universiti Malaysia Perlis, Perlis, Malaysia. The plant leaves were air-dried at room temperature and then crushed into powder using a blender. Infusion method was used for preparation of aqueous plant extracts [23]. In this method, 0.5 g of each type of leaf powder was dissolved in 100 ml of boiling (distilled) water and subsequently allowed to infuse for 15 min without additional heating. Whatman filter papers of pore size 11 μm were used for filtering all samples to obtain the plant extracts and prepare 1, 2, 3, and 4 mg/mL plant extract solutions respectively.

### Preparation of Acarbose (Drug) Sample

Ten Acarbose tablets were weighed, and the average weight was calculated. The tablets were ground into a homogeneous fine powder in a mortar. Then, 50 mg of acarbose powder was dissolved in 50 mL distilled water to obtain 1 mg/mL of acarbose solution.

### Amine Functionalization of MWCNTs

The amine functionalization of MWCNTs with EDA was performed by mixing MWCNTs (200 mg), EDA (20 ml), and NaNO_2_ (200 mg). The mixture was heated for 1 h at 60 °C. The resulting mixture was allowed to cool at room temperature and filtered through a Whatman filter paper. The precipitate was washed several times with DMF and finally with distilled water until any unreacted EDA was removed. Then, the amine-functionalized multi-walled carbon nanotubes (MWCNTs-NH_2_) were dried at 50 °C for 48 h [24, 25].

### Immobilization of Enzyme by Covalent Attachment

AG enzyme was immobilized onto MWCNTs-NH_2_ using EDC-NHS chemistry [26]. A 20-mg MWCNTs-NH_2_ was added into 5 ml MES [2-(*N*-morpholino)ethane sulfonic acid] buffer (100 mM, pH 6.0) containing 100 mM EDC and 100 mM NHS for 6 h at room temperature. The suspension was filtered through Whatman filter paper and rinsed thoroughly with 100 mM MES buffer solution (pH 6.0) and then washed with 0.1 M PB solution (pH 6.0) to remove unreacted excess EDC and NHS. The activated MWCNTs-NH_2_ particles were then redispersed in 5 ml PB solution (100 mM, pH 6.0) containing 0.2 mg/mL AG enzyme for 3 h at 4 °C. After incubation, the suspension was filtered and then rinsed with 100 mM PB solution (pH 7.0) to remove any unbound enzyme. The filtrated product MWCNT enzyme conjugate (MWCNT-AG) was subsequently used to develop the biosensor.

### Measurement of Immobilized AG Enzyme

The immobilized AG enzyme onto MWCNTs was measured by subtracting the amount of enzyme in initial solution from the amount of enzyme both in filtrate and washing solutions after immobilization. The amount of enzyme in the supernatant at each washing was calculated until no enzyme was leached. The percentage of immobilized enzyme was calculated as follows (Eq. (1)) [27].

1 $$ \mathrm{Immobilized}\ \mathrm{enzyme}\ \left(\%\right) = \left(\mathrm{amount}\ \mathrm{of}\ \mathrm{enzyme}\ \mathrm{introduced}\ /\ m\right) \times 100 $$

where *m* is the amount of enzyme in the final solution. The AG enzyme was assayed according to the Sigma’s quality control procedure.

### Preparation of PVA Solution by Freezing–Thawing Process

Aqueous PVA solutions with different concentrations (0.1, 0.2, 0.3, 0.4, 0.5, 0.6 wt%) were heated at 90 °C for 1 h. The solutions were then cooled to room temperature and subsequently left at −20 °C for 12 h. After the freezing, the solutions were allowed to thaw at 4 °C for another 12 h. The freezing–thawing cycle was performed to reinforce of the PVA solution via densification of the macromolecular structure. Moreover, after the freezing–thawing treatment of the PVA solution, a porous structure arises due to gelatin processes [28].

### Biosensor Preparation Using SPCE, PNPG-PVA, and MWCNTs-AG Biocomposite

PNPG was dissolved in PB solution (0.1 M, pH 4.5) and then mixed with freeze-thawed PVA solution (pH 4.5, 0.4 wt%) to prepare 1.0, 2.0, 3.0, 4.0, and 5.0 mM PNPG solution. The PNPG-PVA solution and MWCNTs-AG composite at the ratio of 4:1 % *w*/*w* were mixed together and using a vortex to obtain a homogeneous MWCNTs-AG-PNPG-PVA biocomposite mixture. The MWCNTs-AG-PNPG-PVA biocomposite film was prepared by casting 8 μL of this biocomposite mixture on the SPCEs. The solvent in the mixture was allowed to evaporate at room temperature for 1 h. Afterward, the biosensor was kept at 4 °C prior to use.

### Preparation of PNPG Electrode

PNPG was dissolved in PB solution (0.1 M, pH 7.0) and then mixed with freeze-thawed PVA solution (pH 7.0, 0.4 wt%) to prepare 1.0, 2.0, 3.0, 4.0, and 5.0 mM PNPG solution. The PNPG-PVA solution and MWCNTs at the ratio of 4:1 % *w*/*w* were mixed together using a vortex to obtain a homogeneous MWCNTs-PNPG-PVA composite mixture. The MWCNTs-PNPG-PVA composite (8 μL) was then dropped on the SPCE. The solvent in the mixture was allowed to evaporate at room temperature for 1 h. Then, the modified PNPG electrodes were kept at 4 °C before using.

### Cyclic Voltammetric Measurement Method

CV measurements were performed using the biosensor at a scan rate of 20 mV/s after dropping 50 μL of the PB solution (0.1 M, pH 8.75) with 0.1 M KCl. The voltammograms were recorded as control for a given period at room temperature. Different concentration of medicinal plant extracts (1.0, 2.0, 3.0, 4.0 mg/mL) and commercial antidiabetic drugs were used with the PB solution and recorded voltammograms for a given period to measure the antidiabetic potential of the plants. The deposition potential (Edep) and time (tdep) for each measurements were set to −6.0 V and 15 s, respectively.

### Amperometric Measurement Method

The working potential for the amperometric measurement was determined via CV studies using the developed biosensor and PNPG electrode at a scan rate of 20 mV/s. In this measurement, 50 μL of 0.1 M PB solution (pH 8.75) containing 0.1 M KCl was placed on the biosensor. The voltammograms were recorded at a fixed potential for a given period. Afterward, different concentrations of medicinal plant extracts (1.0, 2.0, 3.0, 4.0 mg/mL) and commercial antidiabetic drugs were used with 0.1 M PB solution (pH 8.75) as inhibitor, and the amperometric signals were recorded. In the same way, the PNPG electrode was used with 0.1 M PB solution pH 7.0 and recorded amperometric signal for PNPG. The Edep and tdep for each measurement were set to −6 V and 15 s, respectively. The measurements were performed at room temperature.

### Spectrophotometric Measurement Method

Colorimetric measurements were performed at room temperature using a Lambda 25 UV–VIS spectrometer (PerkinElmer, USA). In this method, the biosensor was immersed in 0.1 M PB solution (pH 7.5) to ensure a complete reaction between the immobilized AG enzyme and PNPG in the biosensor. After a given period, the biosensor was removed from the PB solution. Then, the absorbance of the released p-NP was measured and subsequently used as the control. Different concentration of medicinal plant extracts (1.0, 2.0, 3.0, 4.0 mg/mL) and commercial antidiabetic drugs were used with the PB solution (pH 7.5), and the released p-NP was measured as before. The antidiabetic potential of medicinal plants and commercial antidiabetic drugs was determined as the difference between the absorbance of the control sample and the actual sample. The absorbance of the released p-NP was measured at 400 nm [29].

### Enzyme Activity Measurement Method

The free and immobilized enzyme activity was measured using PNPG electrode and biosensor by Spectrophotometric method at room temperature. The biosensor was immersed in 0.1 M PB solution of pH 7.5 for a given time to facilitate the complete reaction between the immobilized AG enzyme and PNPG in the biosensor. The PNPG electrode was immersed in AG enzyme (same concentration and amount of a biosensor) solution with 0.1 M PB at pH 7.5 for the same reaction time like biosensor. The released p-NP was measured from both solutions by spectrophotometric method at 400 nm and calculated using the calibration curve of p-NP.

## Results and Discussion

### Cyclic Voltammetric Behavior of the Biosensor

**Fig. 2:**
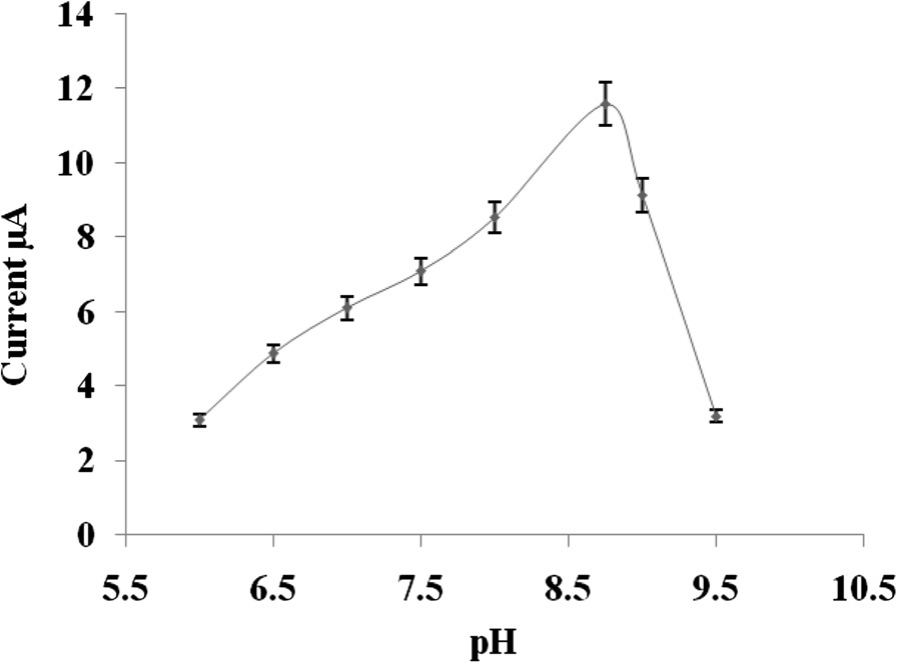
Plotting the different pH of 0.1 M buffer solution with oxidation peak current of released p-NP resulting the reaction between immobilized AG enzyme and PNPG in the biosensor

The pH of the PB solution is a vital parameter to determine the electrocalytic activity of the developed biosensor. AG enzyme (0.2 mg/mL) and PNPG (3.0 mM) were immobilized with PVA solution (0.4 wt%) on the SPCE at pH 4.5 to prevent the premature reaction between the immobilized PNPG and AG enzyme. To determine the biosensor activity, a particular pH of the PB solution is required to facilitate the reaction between the immobilized AG enzyme and PNPG. For this purpose, PB solutions of pH 6.0 to 9.5 were used with 0.1 M KCl solution to determine the optimum reaction pH between the immobilized AG enzyme and PNPG. The CV measurements were performed at scan rate 20 mV/s, and the results show in Fig. 2 that the optimal biosensor electrocatalytic activity was obtained at pH 8.75 of the PB solution.

PVA solution concentration plays an important role to measure the biosensor activity. Different concentrations of PVA solution were used to select a particular concentration of PVA solution for obtaining the maximum biosensor electrocatalytic activity. The maximum electrocatalytic activity of biosensor was achieved with 0.4 wt% PVA solution. Accumulation potential and time are two significant parameters for determination the maximum peak current by CV method. The accumulation potential and time was selected −6.0 V and 15 s respectively for maximum peak current of released p-NP. All measurements were performed at ambient room temperature.

**Fig. 3:**
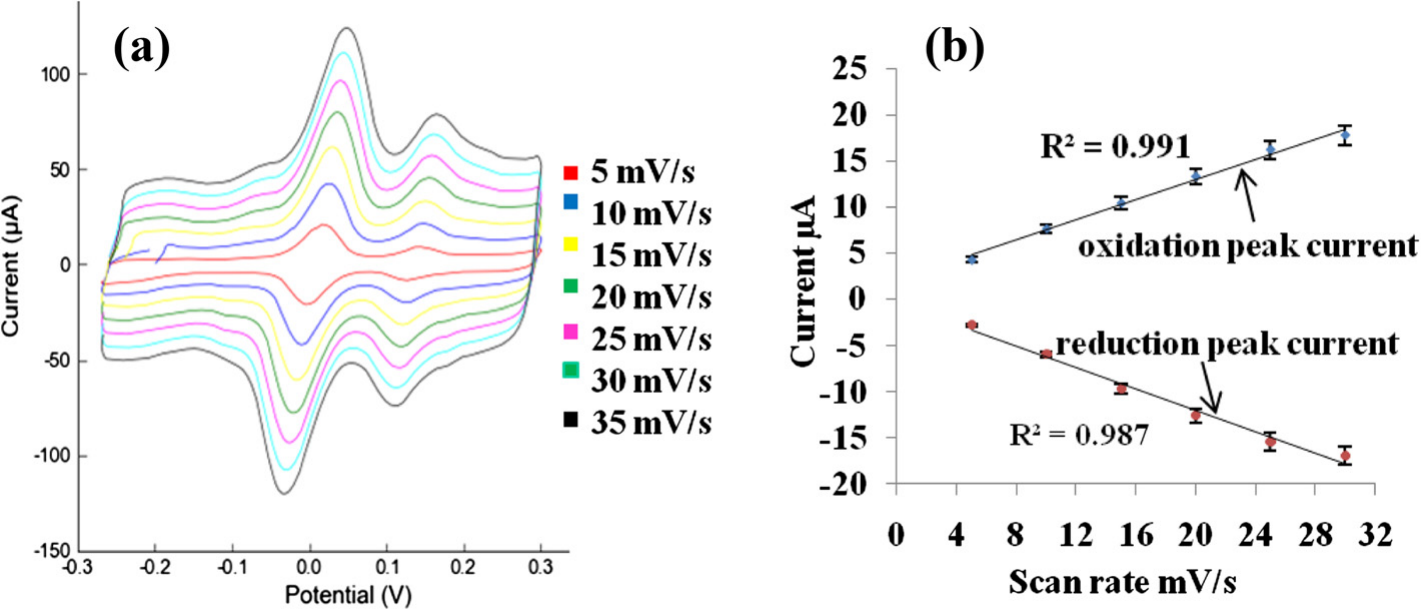
**a** Cyclic voltammograms of the biosensor with 0.1 M PB solution of pH 8.75 at 10 to 60 mV/s scan rate. **b** The corresponding plot of released p-NP oxidation and reduction peak current versus different scan rates

The CV results of the biosensor at different scan rates from 5 to 30 mV/s are shown in Fig. 3a. The corresponding plot in the Fig. 3a shows that the oxidation and reduction peak currents of released p-NP increase linearly with the increase of scan rate from 5 to 30 mV/s. Besides, the redox peak currents are directly proportonal to the scan rate, *v*, which are shown in Fig. 3b. The linear regression equations for the oxidation and reduction peak currents are *I*_pa_ (μA) = 0.547*v* + 2.053 and *I*_pc_ (μA) = −0.584*v* − 0.331 with the correlation coefficients (*R*^2^) of 0.991 and 0.987, respectively, where, *v* is the scan rate. This indicates that the surface reaction of the biosensor follows a typical surface-controlled eletrochemical process [30]. The oxidation and reduction peak are slightly shifted to positive and negative potential with increasing scan rate, indicating the quassi-reversible process [31].

**Fig. 4:**
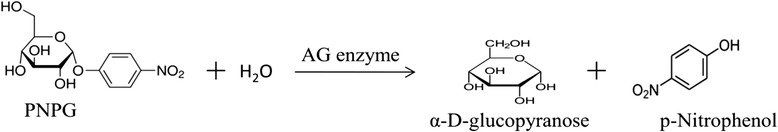
Hydrolysis reaction of PNPG with AG enzyme

**Fig. 5:**
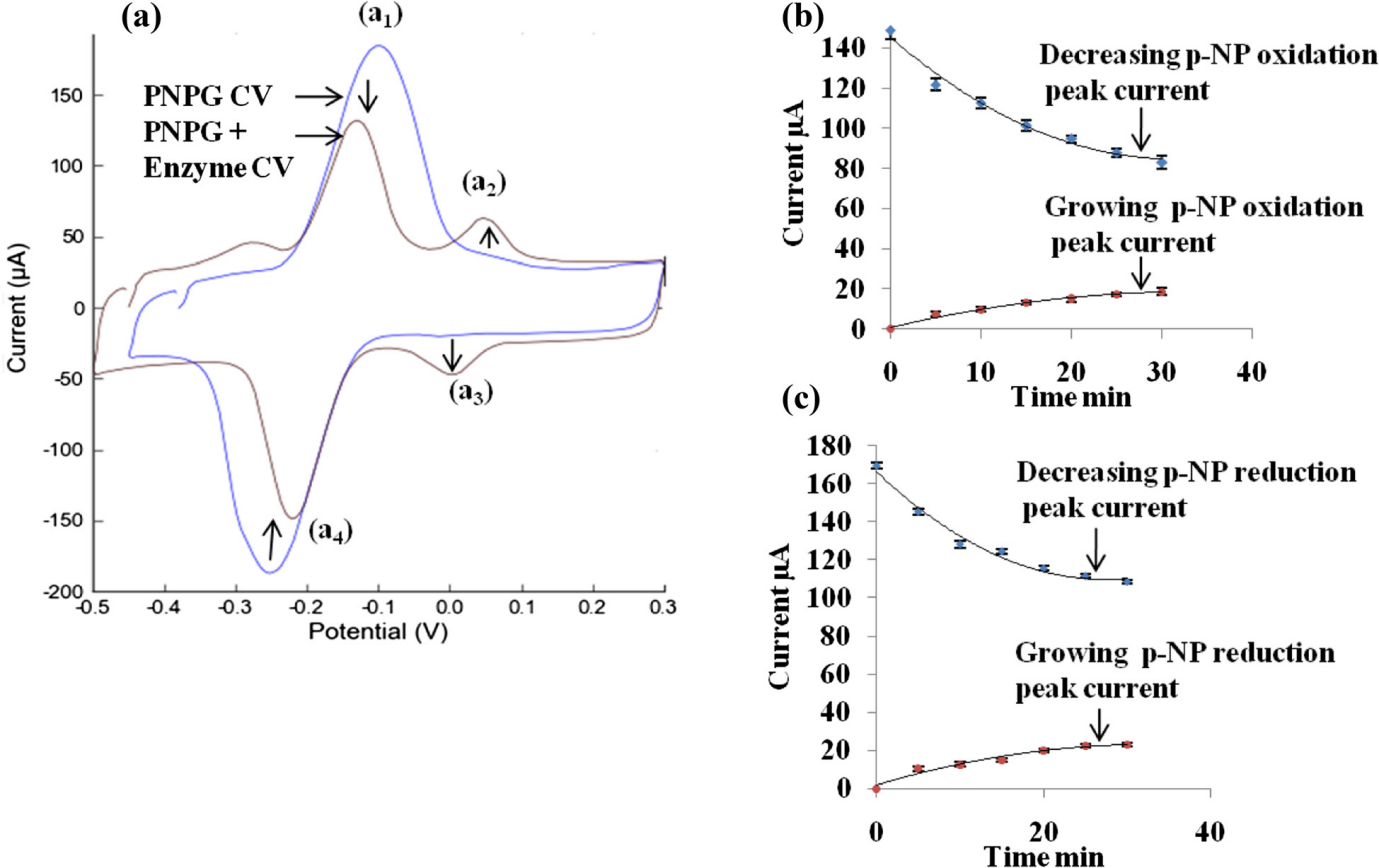
**a** Cyclic voltammograms of PNPG (*red*) and PNPG + enzyme (*blue*) after 20 min in presence of 0.1 M phosphate buffer at pH 6.8 on the SP-CNT electrode, (*a*_*1*_) decreasing attached p-NP oxidation peak, (*a*_*2*_) growing released p-NP oxidation peak, (*a*_*3*_) growing released p-NP reduction peak, (*a*_*4*_) decreasing attached p-NP reduction peak; **b** decreasing and growing oxidation peak currents of the attached and the released p-NP; and **c** decreasing and growing reduction peak currents of the attached and the released p-NP

In the biosensor, PNPG is hydrolyzed by AG enzyme in the presence of PB solution (pH 8.75) to release p-NP as in Fig. 4 [32]. The released p-NP is measured by CV method which is shown in Fig. 5a.

The decreasing PNPG (attached p-NP) oxidation peak current and the growing of released p-NP oxidation peak current are shown in the Fig. 5b, and the decreasing PNPG (attached p-NP) reduction peak current and growing released p-NP reduction peak current are shown in the Fig. 5c as a function of time. Thus, the values of the oxidation or reduction peak currents of the released p-NP can be used to measure the immobilized AG enzyme activity in the biosensor.

### Measurement of the Inhibitory Activities of Medicinal Plant Extracts and the Synthetic Drug (Acarbose) by CV Method

**Fig. 6:**
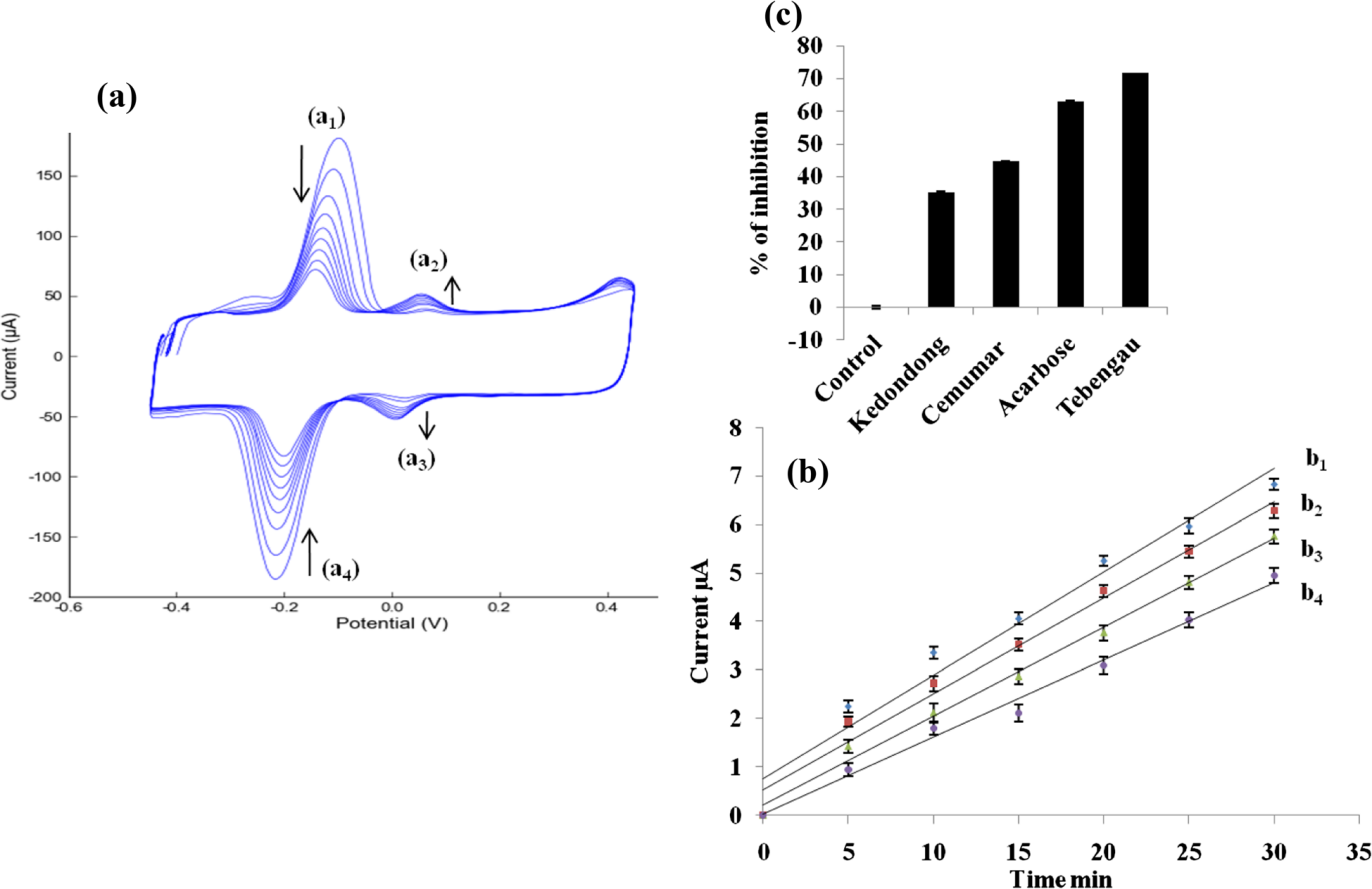
**a** Cyclic voltammograms of biosensor with PB solution (control), (*a*_*1*_) decreasing attached p-NP oxidation peak, (*a*_*2*_) growing released p-NP oxidation peak, (*a*_*3*_) growing released p-NP reduction peak, (*a*_*4*_) decreasing attached p-NP reduction peak. **b** Inhibition potential of (*b*_*1*_)Tebengau, (*b*_*2*_) Acarbose, (*b*_*3*_) Cemumar, and (*b*_*4*_) Kedondong by current as a function of time. **c** % inhibition of Tebengau, Acarbose, Cemumar, and Kedondong

In the presence of medicinal plant extracts as inhibitors, the enzymatic reaction between the immobilized PNPG and AG in the biosensor exhibits a lower peak current of released p-NP. The inhibition potential of the medicinal plant extracts and the commercial drug was determined by comparing their CV responses of the control and sample. The cyclic voltammograms of the biosensor with the control are shown in Fig. 6a. When any medicinal plant extract was added as an inhibitor into the PB solution, the oxidation and reduction peak currents of the released p-NP decreased as compared to those of the control.

The three different medicinal plants extracts (Tebengau, Cemumar, Kedondong) and the commercial drug acarbose (2.0 mg/mL) were used to measure the inhibition potential in the biosensor by the difference of uninhibited and inhibited oxidation or reduction peaks of the released p-NP. The corresponding plots in Fig. 6b show that the inhibited released p-NP by Tebengau is higher than acarbose, Cemumar, and Kedondong. The inhibition percentage of medicinal plants and acarbose in Fig. 6c shows that the percentage inhibition of Tebengau is higher than acarbose, Cemumar, and Kedondong. The percentage of inhibition was calculated following Eq. (1) [33].

2 $$ \%\ \mathrm{of}\ \mathrm{i}\mathrm{nhibition}\kern0.5em =\kern0.5em \frac{\mathrm{ip}/\mathrm{Abs}.\ \mathrm{of}\ \mathrm{control}\ \hbox{--}\ \mathrm{i}\mathrm{p}/\mathrm{Abs}.\ \mathrm{of}\ \mathrm{sample}}{\mathrm{ip}/\mathrm{Abs}.\ \mathrm{of}\ \mathrm{control}}\kern0.5em \times \kern0.5em 100 $$

The inhibition kinetics of the immobilized AG enzyme in the biosensor was investigated in the presence of different concentrations of Tebengau herbal extracts (1, 2, 3, 4 mg/mL) as inhibitors as well as of various PNPG concentrations (1, 2, 3, 4, 5 mM) in the biosensor. The inhibition kinetics was determined with respect to the apparent Michaelis–Menten constant (*K*_m_) which was obtained from the Lineweaver–Burk equation:

3 $$ \frac{1}{V}=\left(\frac{K_{\mathrm{m}}}{V_{\max }}\right)\left(\frac{1}{\left[S\right]}\right)+\frac{1}{V_{\max }} $$

The apparent *K*_m_ represents the kinetics of the reaction between the enzyme and the substrate.

**Fig. 7:**
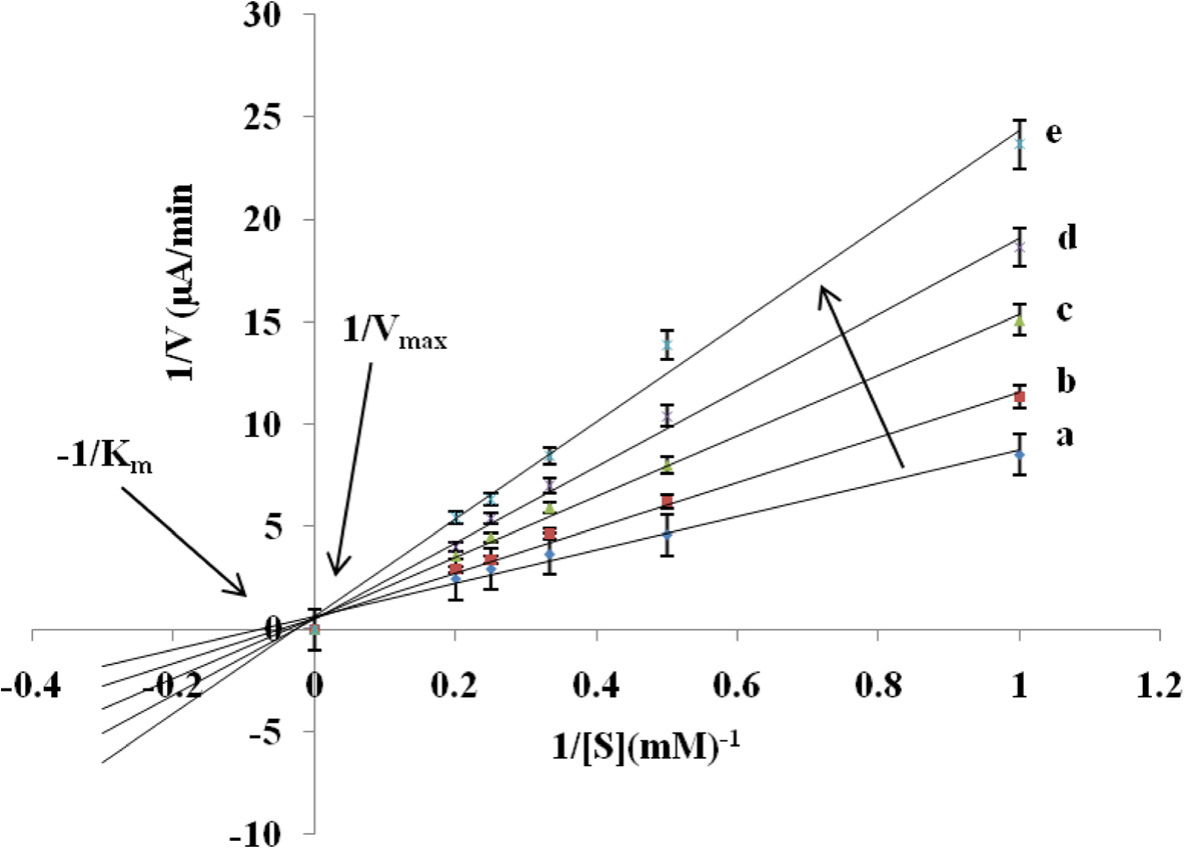
Effects of different concentrations of Tebengau plant extracts on the hydrolysis reaction between the immobilized AG enzyme and PNPG (*p*-nitrophenyl-α-d-glucopyranoside) in the biosensor. **a** Control (PNPG + enzyme). **b** Control + 10 μL (1 mg/mL) Tebengau plant extracts. **c** Control + 10 μL (2 mg/mL) Tebengau plant extracts. **d** Control + 10 μL (3 mg/mL) Tebengau plant extracts. **e** Control + 10 μL (4 mg/mL) Tebengau plant extracts

The Lineweaver–Burk equation plotted in Fig. 7 shows the inhibitory effects of different Tebengau concentrations on the reaction between the immobilized AG enzyme and different PNPG concentrations (substrate) in the biosensor. The *K*_m_ values were calculated using the slopes and intercepts of the current plot vs. different substrate concentrations.

The corresponding results shown in Table 1 indicate that the apparent *K*_m_ increases with increasing Tebengau concentration, whereas *V*_max_ is nearly unchanged. A low *K*_m_ indicates a high affinity of the enzyme for the substrate, whereas a high *K*_m_ indicates a low affinity between the enzyme and the substrate because of the inhibition of enzyme activity by the Tebengau medicinal plant extracts. This type of inhibition is known as competitive inhibition [34] in which both the inhibitor and the substrate are bound to the active side of the enzyme. In this case, the inhibition increases with increasing concentration of the medicinal plant extracts. Moreover, this inhibition can be overcome by using a specific substrate concentration at which *V*_max_ remains unaffected [35]. In the present study, the hydroxyl groups of the phenolic compounds in the medicinal plant extracts compete with the hydroxyl groups of PNPG.

**Table 1:** *K*_m_ and *V*_max_ values at different concentrations of Tebengau plant extracts

Concentration of Tebengau plant extracts (μL)	*K*_m_ value (mM) (apparent)	*V*_max_ value (μA/min)
Control	12.898	1.612
10 μL (1 mg/mL)	19.742	1.785
10 μL (2 mg/mL)	25.791	1.745
10 μL (3 mg/mL)	34.818	1.796
10 μL (4 mg/mL)	38.929	1.639

The above discussion not only confirms the authenticity and reliability of the antidiabetic potential measurement method but also suggests that the modified screen-printed disposable biosensors are suitable for the monitoring of the antidiabetic potential of medicinal plants as well as the synthetic commercial drugs.

### Measurement of the Inhibitory Activities of Medicinal Plant Extracts Using Biosensor Based on Amperometric Responses

**Fig. 8:**
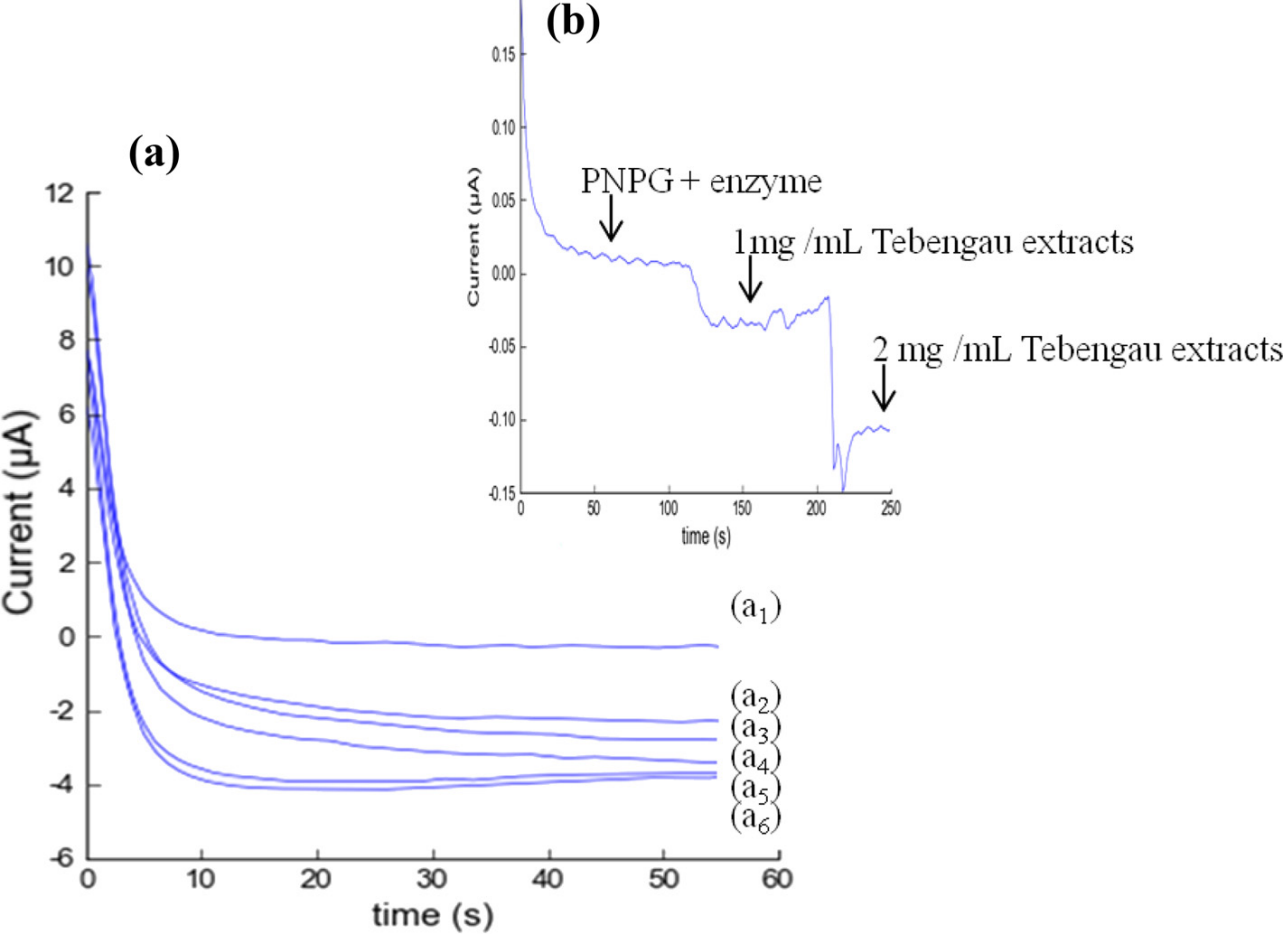
**a** Amperometric responses of (*a*_*1*_) PNPG + enzyme, (*a*_*2*_) PNPG + enzyme + 1.0 mg/mL extracts, (*a*_*3*_) PNPG + enzyme + 2.0 mg/mL extracts, (*a*_*4*_) PNPG + enzyme + 3.0 mg/mL extracts, (*a*_*5*_) PNPG + enzyme + 4.0 mg/mL extracts, and (*a*_*6*_) PNPG. **b** Amperometric response of biosensor with stirring using different concentration Tebengau plant extracts

The inhibitory effects of Tebengau plant extracts (1.0–4.0 mg/mL) on AG enzyme in biosensor were determined amperometrically as shown in Fig. 8a, b). In the absence of medicinal plant extracts, the enzymatic hydrolysis of PNPG occurs in the biosensor without any inhibition. However, in the presence of medicinal plant extracts as inhibitors, the enzyme could not hydrolyze PNPG properly. As a result, the current response of PNPG will be increased. Therefore, the current response increases with the increase of medicinal plant extract concentration until completed inhibition of enzymatic activity. The PNPG electrode was used as the control sample.

### Inhibition Studies of Tebengau Plant Extracts Using Biosensor Via Amperometric, Cyclic Voltammetric, and Spectrophotometric Methods

**Fig. 9:**
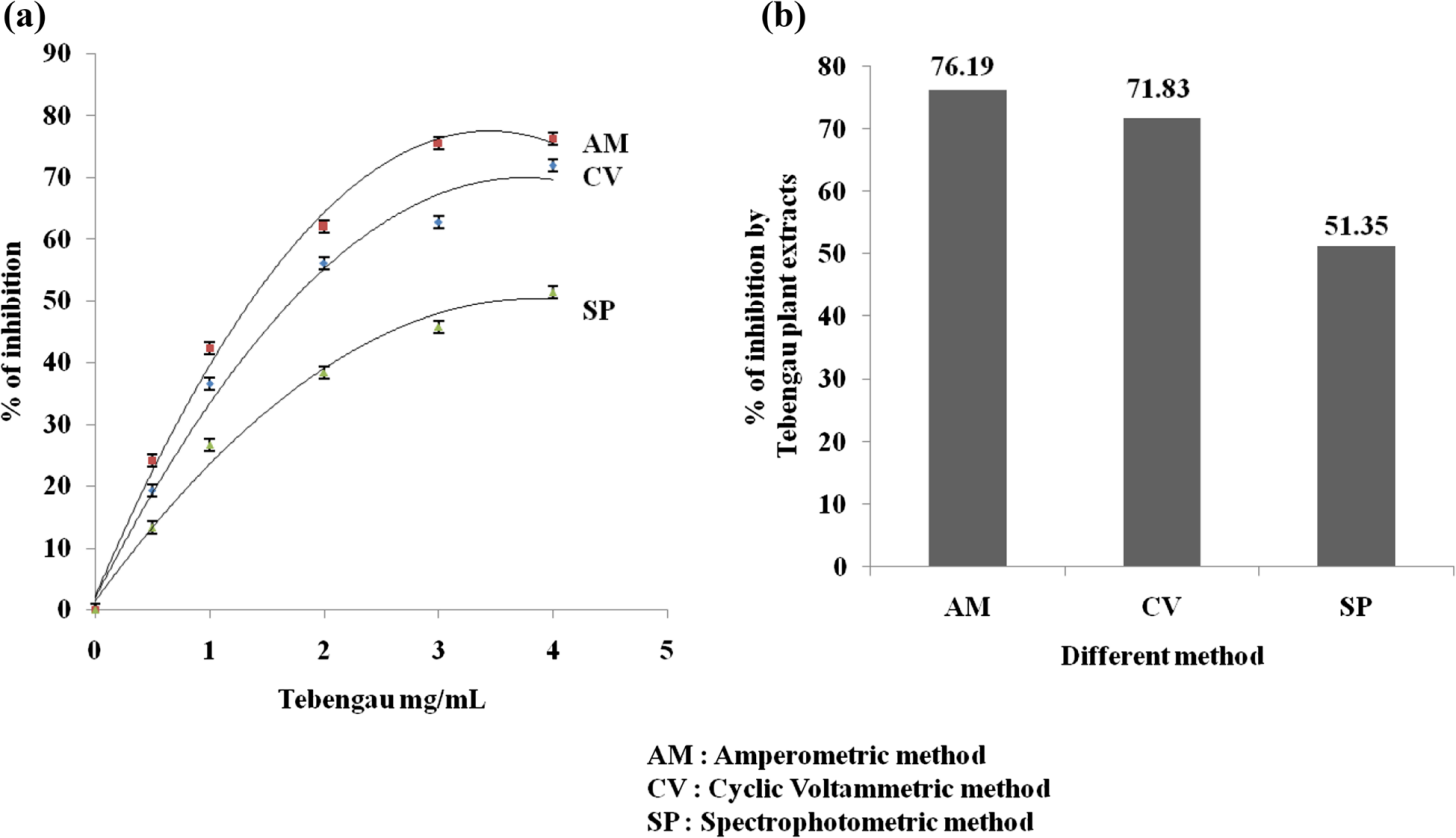
**a** Measurement of percent inhibition by different concentration of Tebengau plant extracts using biosensor via amperometric cyclic voltammetric and spectrophotometric method. **b** Percent inhibition of different method with bar graph

The inhibition of Tebengau plant extracts (10 μL, 2.0 mg/mL) was determined using the biosensor via amperometric, cyclic voltammetric, and spectrophotometric methods. The results show in Fig. 9a, b that the percentage of inhibitions by amperometric, cyclic voltammetric, and spectrophotometric methods are 76.19, 71.83, and 51.35 %, respectively. Therefore, electrochemical methods are more sensitive for measuring the antidiabetic potential of medicinal plants than colorimetric method. The percentage of inhibition was calculated as Eq. (1).

### Analytical Performance of the Biosensor

**Fig. 10:**
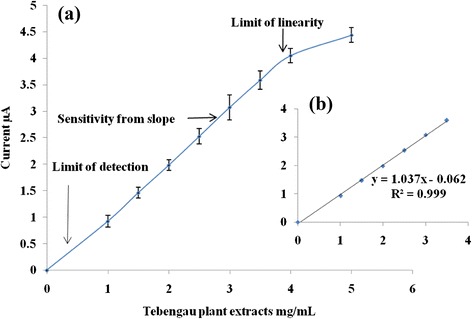
**a** Calibration curve of enzyme activity inhibition by Tebengau plant extracts. **b** Linear range of enzyme activity inhibition by Tebengau plant extracts

A linear relationship was obtained between the inhibition of PNPG and enzyme reaction by different concentration of Tebengau plant extracts in biosensor. A calibration curve of enzyme activity inhibition by Tebengau plant extracts (1.0–5.0 mg/mL) shown in Fig. 10a. The enzyme activity inhibition increases with increasing of plant extracts concentration and the enzyme activity is completely inhibited at high concentration of Tebengau plant extracts. A good linearity was observed using 1.0 to 3.5 mg/mL Tebengau plant extracts with 0.923–4.053 μA response current which is shown in Fig. 10b.

A good sensitivity of the biosensor to detect the inhibition of enzyme activity by plant extracts was observed from the slope of the enzyme activity inhibition calibration curve and it was 1.037 μA/mL plant extracts. The minimum inhibition of enzyme activity was estimated by 0.5 mg/mL of Tebengau plant extracts resulting 0.437 μA current. The biosensor responded very rapidly within 22 s. All analytical performance parameters were studied using five different biosensors by CV method (Table 2).

**Table 2:** Summary of analytical performance of the sensor

Linear range (mg/mL)	R^2^	Sensitivity (μA/mg)	Limit of detection (mg/mL)	Response time (average of 5 times) (sec)
1.0–3.5	0.999	1.037	0.5	22

### Biosensor Reproducibility

**Fig. 11:**
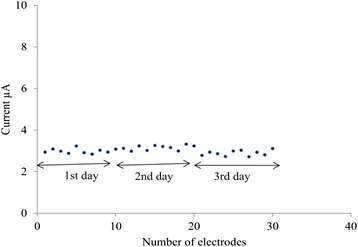
Reproducibility of biosensor

A set of 30 modified, screen-printed biosensors were prepared for three consecutive days using the same composition and procedure. Each day, ten biosensors were used to measure the CV response for a specific time. The CV peak currents varied from 0.5 to 4.9 % at a constant ambient room temperature which is shown in Fig. 11. These variations indicate the reliability of the developed disposable biosensor.

### Storage Effect

**Fig. 12:**
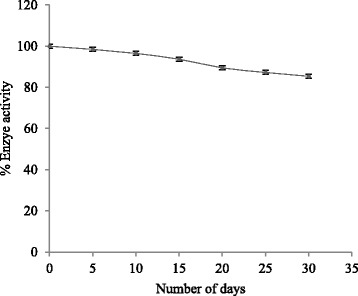
Storage effect of biosensor

A total of 30 modified screen-printed biosensors with the same compositions were prepared and subsequently stored at 4 °C. The CV responses of three electrodes were measured every 5 days within a period of 30 days. Approximately 82.16 % of the initial response current was retained after 30 days which is shown in Fig. 12. This stability indicates the suitability of the biosensor fabrication method and that no damage occurred on the immobilized enzyme and PNPG. These observations indicate the high stability of the biosensor as a result of the covalent attachment of the enzyme.

### Enzyme Immobilization Effect

The activity of free and immobilized enzyme was compared depending on the amount of released p-NP from immobilized PNPG both in the PNPG electrode and biosensor catalyzed by free AG enzyme and immobilized AG enzyme. The results showed that the immobilized AG enzyme catalyzed the immobilized PNPG and liberated p-NP from the biosensor 27.8 % less than the free AG enzyme which liberated p-NP from PNPG electrode. It is mentioned that the immobilized AG enzyme lost 27.8 % catalyzing activity compared to the free AG enzyme due to immobilization on the SP-CNTs.

## Conclusions

In this study, a practical and first response biosensor for measuring the antidiabetic potential of medicinal plants has been introduced. The biosensor carries some advantages with the following. Firstly, it is insensitive to the color of the medicinal plant extracts. Secondly, the response time is 22 s. Hence, it is fast and suitable for screening of antidiabetic potential of the medicinal plants. Moreover, it is disposable and could be used on-site for routine quality control. Additionally, this biosensor as with many other biosensors is easy to operate. The only downside of the developed biosensor is the requirement to operate in a particular buffer solution. However, for practical purposes, the buffer can be supplied with the biosensor. Apart from that, the preparation of the biosensor is simple and free from toxic chemicals and thus can be regarded as an environmentally friendly biosensor.
